# Identification of hereditary breast and ovarian cancer germline variants in Granada (Spain): NGS perspective

**DOI:** 10.1007/s00438-022-01891-5

**Published:** 2022-04-22

**Authors:** María Molina-Zayas, Carmen Garrido-Navas, Jose Luis García-Puche, Julian Barwell, Susana Pedrinaci, Margarita Martínez Atienza, Susana García-Linares, Tomás de Haro-Muñoz, Jose Antonio Lorente, M. Jose Serrano, Antonio Poyatos-Andújar

**Affiliations:** 1 UGC de Laboratorios, Hospital Universitario Clínico San Cecilio, Avda de la Investigación s/n, 18016 Granada, Spain; 2 Genetics Department, Faculty of Sciences, Universidad de Granada, 18071 Granada, Spain; 3 CONGEN, Genetic Counselling Services, C/Albahaca 4, 18006 Granada, Spain; 4 Oncology Department, Vithas Granada Hospital, Avda de Santa María de La Alhambra, Granada, Spain; 5 Leicestershire Clinical Genetics Service, University Hospitals of Leicester NHS Trust, Leicester, UK; 6 UGC de Laboratorios, Hospital Universitario Virgen de Las Nieves, Avda de Las Fuerzas Armadas, 2, 18014 Granada, Spain; 7 Legal Medicine Department, Medicine School, Universidad de Granada, 18016 Granada, Spain; 8 Department of Medical Oncology, Bio-Health Research Institute (Instituto de Investigación Biosanitaria Ibs GRANADA), Hospital Universitario Virgen de Las Nieves Granada, University of Granada, Granada, Spain; 9 Department of Pathological Anatomy, Faculty of Medicine, Campus de Ciencias de la Salud, University of Granada, Granada, Spain

**Keywords:** Hereditary breast and ovarian cancer (HBOC), *BRCA1*, *BRCA2*, NGS, Multigene panel

## Abstract

The aim of this study was to assess the prevalence of germline variants in cancer-predisposing genes by either targeted (*BRCA1/2*) or multigene NGS panel in a high-risk Hereditary Breast and Ovarian Cancer (HBOC) cohort. Samples from 824 Caucasian probands were retrospectively collected and the impact of genetic diagnosis and genetic variants epidemiology in this cohort was evaluated. Performance of risk-reducing prophylactic measures, such as prophylactic mastectomy and/or prophylactic oophorectomy, was assessed through clinical follow-up of patients with a positive genetic result. Pathogenic variants predisposing to HBOC were identified in 11.9% (98/824) individuals at *BRCA2* (47/98), *BRCA1* (24/98), *PALB2* (8/51), *ATM* (7/51), *CHEK2* (6/51) *MSH6*, (2/51), *RAD51C* (2/51) and *TP53* (2/386). Of them, 11 novel pathogenic variants and 12 VUS were identified, characterized, and submitted to ClinVar. Regarding clinical impact, the risk of developing basal or Her2 breast cancer was increased 15.7 times or 37.5 times for *BRCA1* and *MSH6* pathogenic variants respectively. On the contrary, the risk of developing basal or luminal A breast cancer was reduced to 81% or 77% for *BRCA2* and *BRCA1* pathogenic variants, respectively. Finally, 53.2% of individuals testing positive for class IV/V variants underwent prophylactic surgery (mastectomy, oophorectomy or both) being significantly younger at the cancer diagnosis than those undertaking prophylactic measures (*p* = 0.008). Of them, 8 carried a pathogenic/likely pathogenic variant in other genes different from *BRCA1* and *BRCA2*, and the remaining (46.7%) decided to continue with clinical follow-up. No differences in pathogenicity or risk of developing cancer were found for *BRCA1/2* between targeted and multigene sequencing strategies; however, NGS was able to resolve a greater proportion of high-risk patients.

**Supplementary Information:**

The online version contains supplementary material available at 10.1007/s00438-022-01891-5.

## Introduction

Breast and ovarian cancer are the most frequent cancer types affecting women worldwide (Bray et al. 2018). Beyond sporadic cancer, there is a critical population of individuals who carry mutations in some cancer-predisposing genes thus being at higher risk of developing those malignancies. Particularly, from 5 to 10% of all breast cancer (BC) and up to 25% of all ovarian cancer patients (OC) have a genetic predisposition, constituting a medical entity named *Hereditary breast and ovarian cancer syndrome* (HBOC) with mainly autosomal inheritance, incomplete penetrance and variable expressivity (Balmaña et al. 2011). More than two decades ago, the *BRCA1* and *BRCA2* susceptibility genes were discovered and cumulative risks of developing cancer were estimated. In female patients who carry a deleterious variant in *BRCA1*, the risk of developing BC at 70 years is 60% and for OC of 59%. Regarding *BRCA2*, the cumulative risk for BC is 55% and for ovarian cancer is 17% at the age of 70 (Mavaddat et al. 2013). Presence of pathogenic/likely pathogenic variants in these genes makes it possible to improve the follow-up of carrier patients through two strategies: early detection using imaging tests (MRI and mammograms) or the use of preventive surgery (mastectomy or oophorectomy) to reduce the risk of cancer development (González-Santiago et al. 2019). Yet, *BRCA1/2* was found mutated in only 25% of the HBOC cases, remaining the genetic predisposition of a great proportion of high-risk individuals undisclosed (Schubert et al. 2019).

In our Hospital until mid-2017, molecular diagnosis of HBOC was solely based on the identification of variants in *BRCA1* and *BRCA2* using Sanger sequencing and MLPA analysis. In recent years, the development of second-generation sequencing (NGS) allowed to include other genes related to a higher risk of developing HBOC beyond *BRCA1/2*, improving performance and response time (Pinto et al. 2016). NGS multigene panels are being implemented in carrier screening programs (Castéra et al. 2014; Coppa et al. 2018) having both advantages and disadvantages: the benefits of multiplexing capabilities, reduction of costs, and identification of novel variants need to be balanced with the challenges of identifying an increased number of variants of uncertain significance (VUS), especially related with racial bias of current population studies (Kurian et al. 2018; Li et al. 2020).

It was recently suggested that multigene panels might benefit not only high-risk but also individuals not meeting referral criteria for testing (Beitsch et al. 2019; LaDuca et al. 2020). In addition, the identification of actionable variants in genes predisposing to HBOC could well lead to an improved implementation in preventive approaches, screening, surveillance and treatment as we move towards *Precision Medicine* and *Cancer Interception* (Blackburn 2011; Serrano et al. 2020).

Multiple prevalence studies on genetic variants in *BRCA1/2* related to HBOC were carried out in different regions of Spain (Infante et al. 2006; Miramar et al. 2008; Blay et al. 2013; Juan et al. 2013; Gabaldó Barrios et al. 2017; Ruiz De Sabando et al. 2019; Pajares et al. 2018). However, the south part of Spain is slightly under-represented with only one study (Pajares et al. 2018) on *BRCA1/2* which may be relevant given the epidemiological history of the country. Furthermore, none of the above-mentioned studies uses NGS to assess the variant status of other genes beyond *BRCA1/2*.

The main goals of this study were to evaluate our cancer registry to assess the prevalence of germline variants in cancer-predisposing genes besides *BRCA1/2* in Granada’s high-risk HBOC population and to follow-up patients who had a pathogenic (class V) or likely pathogenic (class IV) variant to assess clinical impact regarding prophylactic surgery (either mastectomy, oophorectomy, or both) and periodic follow-up.

## Patients and methods

### Patient recruitment and study design

This retrospective cohort study includes 824 high-risk patients for hereditary breast and/or ovarian cancer (HBOC) referred to the Genetic Counselling Units at two University Hospitals in Granada. The inclusion criteria were defined according to the Spanish Society of Medical Oncology (SEOM) (Graña et al. 2011) (Supplementary table 1). Fulfilling at least one criterion was sufficient to be included in the study, independently on whether more criteria were also fulfilled. Additionally, 294 patients not fulfilling any criteria defined above (mainly due to lack of family information) were also included. The whole cohort consisted of breast cancer (*n* = 650) and ovarian cancer (*n* = 66) patients as well as high-risk cancer-free individuals (*n* = 108). Median age of onset for breast cancer was 45.6 ± 10.9 (range 21–90) and 49.2 ± 11.7 (range 25–70) for ovarian cancer (Table 1). This study was approved by the ethical committee and informed consent was obtained for all patients. Histological cancer subtypes were assessed at the Pathology Unit after solid biopsy followed by tissue immunostaining. Molecular subtypes for BC were referred as luminal A (ER^+^ and/or PR^+^, HER2^−^), luminal B (ER^+^ and/or PR^+^, HER2^+^), HER2 positive (ER^−^ and PR^−^, HER2^+^) and basal (ER^−^, PR^−^ and HER2^−^). Regarding OC, serous, endometrioid, mucinous and epithelial subtypes were characterized in 69.7% of the population. Full histological characterization of the whole cohort could not be assessed in 11.8% of the samples referred as *unk* (unknown). Patients were followed up from the date of testing. For patients with a negative result (either class I, II or III), no further action was taken. Patients with a positive result (class IV or V) were divided into those undergoing risk-reducing preventive surgery (mastectomy, oophorectomy or both) and those who decided only to attend specific check-ups and controls.

**Table 1:** Clinic-pathological and genetic characteristics of the study cohort

	*N*	(%)	Age cancer	Range	Age test	Range	Class I/II (%)	Class III (%)	Class IV/V (%)
Whole cohort	824	(100)	50.1	(21–90)	50.7	(18–92)	625 (75.8)	101 (12.3)	98 (11.9)
Female	800	(97.1)	45.5	(21–82)	50.3	(18–92)	604 (75.5)	101 (12.6)	95 (11.9)
Male	24	(2.9)	60.3	(36–90)	65.6	(37–92)	21 (87.5)	0	3 (12.5)
Tumor type									
Breast cancer	650	(78.9)	45.6	(21–90)	51.1	(25–92)	487 (74.9)	86 (13.2)	77 (11.9)
Female	629	(96.8)	45.1	(21–82)	50.6	(25–92)	469 (74.5)	86 (13.7)	74 (11.8)
Male	21	(3.2)	60.3	(36–90)	66.3	(37–92)	18 (85.7)	0	3 (14.3)
Ovarian cancer (female)	66	(8.0)	49.2	(25–70)	56	(27–86)	49 (74.2)	4 (6.1)	13 (19.7)
Unaffected individuals	108	(13.1)	N/A	N/A	44.6	(18–71)	89 (82.4)	11 (10.2)	8 (7.4)
Female	105	(97.2)	N/A	N/A	44.53	(18–71)	86 (81.9)	11 (10.5)	8 (7.4)
Male	3	(2.8)	N/A	N/A	50.7	(50–52)	3 (100)	0	0
Histological subtype*									
Breast cancer	650	(100)	45.6	(21–90)	51.1	(25–92)	487 (74.9)	86 (13.23)	77 (11.9)
Basal	103	(15.8)	44.8	(27–75)	49.2	(28–81)	75 (72.8)	12 (11.7)	16 (15.5)
Luminal A	324	(49.8)	46.37	(25–82)	50.1	(25–86)	239 (73.8)	48 (14.8)	37 (11.4)
Luminal B	99	(15.2)	45.1	(27–74)	51.6	(30–76)	74 (74.7)	13 (13.1)	12 (7.1)
HER2 positive	27	(4.16)	42.6	(21–59)	46	(27–60)	22 (81.5)	3 (11.1)	2 (7.4)
Unknown	97	(14.9)	45.3	(23–90)	57.6	(29–92)	77 (79.4)	10 (10.3)	10 (9.3)
Ovarian cancer	66	(100)	49.2	(25–70)	56	(27–86)	49 (74.2)	4 (6.1)	13 (19.7)
Endometrioid	6	(9.1)	49.8	(33–65)	57	(43–73)	5 (83.3)	0 (0)	1 (16.7)
Mucinous	4	(6.1)	48.0	(36–62)	55.7	(46–72)	3 (75)	0 (0)	1 (25)
Epithelial	5	(7.6)	54.2	(35–65)	56.2	(36–72)	3 (60)	1 (20)	1 (20)
Serous	31	(47.0)	50.2	(25–70)	54.9	(27–86)	21 (67.8)	1 (3.2)	9 (29)
Others	7	(10.6)	46.9	(36–62)	53	(39–74)	5 (71.4)	2 (28.6)	0 (0)
Unknown	13	(19.7)	46.1	(30–66)	59.6	(44.73)	12 (92.3)	0 (0)	1 (7.7)
Genetic counselling inclusion criteria									
1 familiar cancer (independent on FH)	177	(21.5)	36.9	(21–75)	43.9	(25–82)	130 (73.4)	15 (8.5)	32 (18.1)
Synchronic or metachronic BC and OC in the same individual	12	(6.8)	53.5	(36–75)	65.6	(45–82)	9 (75)	0 (0)	3 (25)
BC < 35 years	96	(54.2)	31	(21–34)	37.8	(25–81)	69 (71.8)	10 (10.4)	17 (17.7)
Bilateral BC, when the first was diagnosed < 40 years old	11	(6.2)	36.8	(35–39)	51.4	(37–67)	9 (81.8)	1 (9.1)	1 (9.1)
Triple negative < 50 years	45	(25.4)	41.6	(35–49)	47	(36–65)	36 (80)	4 (8.9)	5 (11.1)
High-grade serous papillary OC	13	(7.3)	49.6	(27–65)	52.8	(27–73)	7 (53.8)	0 (0)	6 (46.2)
2 familiar cancers (first-degree relatives and in the same family branch)	242	(29.4)	44.1	(25–90)	50	(36–92)	172 (71.1)	37 (15.3)	33 (13.6)
Bilateral BC diagnosed before 50 years old	4	(1.6)	46.5	(43–49)	60.7	(48–89)	3 (75)	0 (0)	1 (25)
1 BC in a male and BC/OC in a female of the family	7	(2.6)	66.3	(46–90)	69.6	(48–92)	5 (71.4)	0 (0)	2 (28.6)
BC and OC	24	(9.9)	48.9	(25–70)	55	(36–86)	17 (70.8)	3 (12.5)	4 (16.7)
2 BC diagnosed before 50 years old	207	(85.5)	42.7	(35–49)	48.6	(36–73)	147 (71)	34 (16.4)	26 (12.6)
≥ 3 BC and/or OC in the family (independently on age)	111	(13.5)	57.8	(50–82)	60.7	(43–86)	80 (72.1)	18 (16.2)	13 (11.7)
Other (no SEOM criteria)	294	(35.6)	49.4	(30–79)	52.5	(18–92)	242 (82.3)	31 (10.5)	21 (7.1)
Unaffected individuals (unaffected index cases tested because of their family history)		108	N/A	N/A	44.6	(18–71)	89 (82.4)	11 (10.2)	8 (7.4)
BC with family history		65	53.7	(38–79)	58.1	(39–81)	53 (81.5)	6 (9.2)	6 (9.2)
BC without family history		76	44.8	(35–73)	50.7	(29–91)	65 (85.5)	10 (13.2)	1 (1.3)
OC with family history		1	50	N/A	53	N/A	1 (100)	0 (0)	0 (0)
OC without family history		16	48.1	(30–68)	56.9	(39–73)	13 (81.2)	1 (6.3)	2 (12.5)
BC/OC unknown family history		28	53.3	(30–72)	61.3	(42–92)	22 (78.6)	3 (10.7)	3 (10.7)

Class I/II includes benign and likely benign variants, Class III are variants of uncertain significance and Class IV/V are pathogenic/likely pathogenic

*N* number, *N/A* non-applicable, *FH* family history, *BC* breast cancer, *OC* ovarian cancer, *%* percentage

### Genetic testing

As genetic testing became more widely implemented within the clinical routine, a transition between targeted sequencing to multigene panel testing was done. Thus, this cohort study includes 824 individuals that were either analyzed by targeted sequencing for *BRCA1/2* (438/824; 53%) or by multigene panel analysis (386/824; 47%). Copy number variation (CNV) analyses to detect small insertion/deletions on *BRCA1/2* were performed by multiplex ligation-dependent probe amplification (MLPA) following the laboratory standard operating procedures. Targeted sequencing for point mutations at *BRCA1/2* was performed using standard Sanger sequencing. Next-generation sequencing (NGS) was performed using a 16 multigene panel (*Hereditary cancer solution* by SOPHiA GENETICS): *ATM, BRCA1, BRCA2, BRIP1, CDH1, CHEK2, MLH1, MSH2, MSH6, PALB2, PMS2, PTEN, RAD51C, RAD51D, STK11*and *TP53* in a MiSeq System (Illumina) at the University Hospitals of Granada following the manufacturer's protocol. This panel covers the coding regions and splicing junctions (± 5 bp) of the genes understudy with a high-confidence calling of SNVs, Indels and CNVs in all genes of the panel. The analysis considers a minimum coverage of ≥ 50 × and an alternative allele with coverage greater than 20. Sequencing was done in a MiSeq (Illumina Inc) and bioinformatics analysis and variants annotation was performed using the SOPHiA DDM 5.8.0.3 software (human reference genome GRCh37/hg19).

### Data analyses

Variant pathogenicity and clinical classification were carried out in accordance with a five-tier system of classifications for variants of the American College of Medical Genetics and Genomics (ACMG): class V pathogenic, class IV likely pathogenic, class III variant of uncertain significance, class II likely benign and class I benign (Richards et al. 2015) using the main databases: Human Gene Mutation Database Professional (HGMD); Leiden Open Variation Database (LOVD); Universal Mutation Database (UMD); Clinical Variation Database (ClinVar); Breast Cancer Information (BCI) and Exome Aggregation Consortium (ExAC). All pathogenic or likely pathogenic variants identified were confirmed by Sanger sequencing or MLPA. For pathogenicity evaluation of the variants of uncertain significance (VUS), different in silico prediction software based on supervised-learning methods were used: 1-SIFT; Poliphen-2; and Mutation Taster (all of which are integrated into the SOPHiA DDM 5.8.0.3 software). If a pathogenic/likely pathogenic variant was identified, no subsequent VUS was reported. In our study, we did not detect more than one pathogenic/likely pathogenic variant.

Descriptive analyses include absolute and relative percentages for categorical variables, mean and standard deviation for continuous normal variables, and median and interquartile range for continuous non-normal variables. For evaluating the association between categorical variables, Chi-square test with simulated *p* value (simulated *p* value because of low expected frequencies in cells in contingency tables) was used. The selected alpha is set to 0.05 (*p* values equal or lower than this one are considered significant). For continuous variables, the association was evaluated using *t* test or Mann–Whitney–Wilcoxon test, or one way or ANOVA Kruskall–Wallis test for more than two groups, depending on normality assumptions. When more than two groups were evaluated, the *p* value for pairs comparisons was corrected using the Holms method for avoiding increasing type I error due to multiple test. Univariate odds ratio with 95% confidence interval were estimated to measure association. When cell frequencies were below one, Haldane–Anscombe correction was applied for allowing the estimation of the confidence interval. For the estimation of adjusted OR, logistic regression models were used. Discrimination ability of logistic models was estimated through the concordance *C* index and Sommer’s *D*. The *C* index takes values between 0 and 1 (the closer to one the greater discrimination capacity). Supplementary table 2 gives a detailed analysis of OR calculations.

## Results

The majority of probands (650/824) had invasive breast cancer (BC) whereas only 8.0% (66/824) had ovarian cancer (OC) (Table 1). The remaining (108/824) were cancer-free individuals but referred as high-risk following the inclusion criteria (Supplementary Table 1). Most BC patients were females with similar distribution for the cancer-free cohort. Predominant histological subtype for BC was luminal A (324/650) whereas luminal B (99/650), and basal subtype were less prevalent (103/650), followed by HER2 positive (27/650). Regarding OC, the majority (31/66) had serous histological subtype followed by endometrioid disease (6/66).

Class IV/V variants were identified in 98/824 (11.9%) patients, being more frequent in OC (13/66) than in BC (78/650). Additionally, actionable pathogenic variants were detected in 8/108 unaffected patients. The most prevalent pathogenic variants, as half population was only tested for those two genes, were found in *BRCA2* (47/98) and *BRCA1* (24/98). In patients with breast cancer, 23.4% (18/77) of the pathogenic/likely pathogenic variants were found in *BRCA1* whereas 48.1% (37/77) were located in *BRCA2*. Regarding ovarian cancer, pathogenic mutations were found in 38.5% (5/13) and 53.8% (7/13) for *BRCA1* and *BRCA2* respectively*.*

**Fig. 1:**
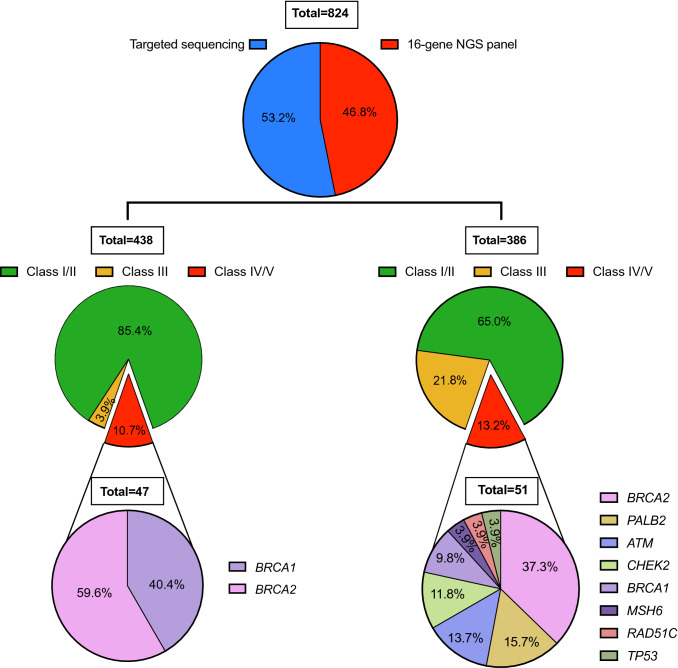
Pie charts describing study design and population distribution for a different type of genetic variants. Class I/II refer to benign and likely benign variants; Class III refers to variants of uncertain significance (VUS) and Class IV/V refer to likely pathogenic and pathogenic variants

Furthermore, of the 51 class IV/V variants identified using the multigene panel, 25 were identified in other genes different from *BRCA1/*2: *PALB2* (8/51), *ATM* (7/51), *CHEK2* (4/51), *MSH6* (2/51), *TP53* (2/51) and *RAD51C* (2/51) (Fig. 1). Results from the multigene panel were compared with results obtained by Sanger sequencing to elucidate the clinical impact of using NGS panels instead of targeted sequencing. When comparing only *BRCA1/2* outcomes, no significant differences were identified regarding cancer occurrence (OR 0.85 [0.21–2.85]) or variant pathogenicity (OR 1.05 [0.51–2.19]). However, when considering all genes included in the multigene panel, significant differences were observed regarding variant pathogenicity (*p* < 0.0001). Thus, the multigene panel was able to significantly resolve more high-risk patients (Supplementary Fig. 1.A) and this was solely dependent on the inclusion of additional genes as no significant differences for *BRCA1/2* outcomes were observed between the two technologies (Supplementary Fig. 1.B). However, not only the number of patients with class IV/V variants increase when using NGS (from 10.7 to 13.2%), but also and more significantly, the number of patients with reported variants of uncertain significance (VUS) increased from 3.9 to 21.8% (for targeted and NGS respectively) (Fig. 1). All pathogenic variants identified in this study are shown in Table 2.

**Table 2:** Pathogenic variants identified in this study

Gene	Nucleotide change	Protein change	dbSNP	Frequency (%)	Cancer type	Frequency in Spanish population (%)	Freq. in ExAC
*BRCA1*	c.68_69delAG	p.Glu23Valfs	rs80357914	4/24 (16)	BC (2), OC (1), Ui (1)	20/155 (12.9) (Juan et al. 2013), 10/60 (16.7) (Gabaldó Barrios et al. 2017), 1/8 (12.5) (del Manzanares Campillo et al. 2018) (12.5) (Gabaldó Barrios et al. 2017; del Manzanares Campillo et al. 2018)	0.00024
	c.3770_3771delAG	p.Glu1257Glyfs	rs80357579	4/24 (16)	BC	11/155 (7.1) (Juan et al. 2013), 1/60 (1.7) (Gabaldó Barrios et al. 2017), 4/50(8) (Pajares et al. 2018), 1/2(50) (Hernan et al. 2015)	0.000008
	c.211A>G	p.Arg71Gly	rs80357382	3/24 (12)	BC	4/155 (2.6) (Juan et al. 2013), 4/60 (6.7) (Gabaldó Barrios et al. 2017), 1/50 (2) (Pajares et al. 2018)	N/A
	c.5123C>A	p.Ala1708Glu	rs28897696	2/24 (8)	BC (1), OC (1)	16/155 (10.3) (Juan et al. 2013), 8/60 (13.3) (Gabaldó Barrios et al. 2017), 5/50 (10) (Pajares et al. 2018) 1/8 (12.5) (del Manzanares Campillo et al. 2018)	N/A
	Del exon 22	N/A	N/A	2/24 (8)	BC	5/1520 (0.33) (Hendrickson et al. 2005)	N/A
	c.5030_5033delCTAA	p.Thr1677Ilefs	rs80357580	1/24 (4)	BC	1/155 (0.7) (Juan et al. 2013)	N/A
	c.1612C>T	p.Gln538Ter	rs80356893	1/24 (4)	BC	–	N/A
	c.302-1G>A	N/A	rs80358116	1/24 (4)	BC	–	N/A
	c.4406dupC	p.Glu1470Argfs	NOVEL	1/24 (4)	BC	–	N/A
	c.470_471delCT	p.Ser157Terfs	rs80357887	1/24 (4)	OC	–	N/A
	c.5078_5080delCTG	p.Ala1693del	rs80358345	1/24 (4)	OC	–	N/A
	c. 83_84delTG	p.Leu28Argfs	rs80357728	1/24 (4)	BC	–	N/A
	c.5266dupC	p.Gln1756Profs	rs80357906	1/24 (4)	BC	1/8 (12.5) (del Manzanares Campillo et al. 2018)	N/A
	c.70_73dup	p.Pro25Leufs	rs397509310	1/24 (4)	BC	–	N/A
*BRCA2*	c.3922G>T	p.Glu1308Ter	rs80358638	5/47 (10.6)	OC (2), BC (3)	7/155 (4.5) (Juan et al. 2013), 1/47 (2) (Gabaldó Barrios et al. 2017), 1/17 (5.9) (Macias 2016)	N/A
	c.3264dupT	p.Gln1089Serfs	rs80359380	4/47 (8.5)	BC	4/47 (8.5) (Gabaldó Barrios et al. 2017), 1/16 (6.25) (del Manzanares Campillo et al. 2018)	0.000008
	c.5720_5723delCTCT	p.Ser1907terfs	rs80359530	4/47 (8.5)	BC (3), Ui (1)	2/155 (1.3) (Juan et al. 2013), 1/16 (6.25) (del Manzanares Campillo et al. 2018), 6/70 (8.6) (Pajares et al. 2018)	N/A
	c.1792delA	p.Thr598Hisfs	rs886040389	2/47 (4.3)	BC (1), OC (1)	1/6 (16.7) (Hernan et al. 2015)	N/A
	c.5576_5579delTTAA	p.Ile1859Lysfs	rs80359520	2/47 (4.3)	BC	1/20 (3.7) (Blay et al. 2013), 3/155 (1.9) (Juan et al. 2013)	0.000033
	c.6024dupG	p.Gln2009Alafs	rs80359554	2/47 (4.3)	BC	2/15 (13.3) (Ramírez-Calvo et al. 2019)	N/A
	c.6275_6276delTT	p.Leu2092Profs	rs11571658	2/47 (4.3)	BC	6/155 (3.9) (Juan et al. 2013), 4/16 (25) (del Manzanares Campillo et al. 2018), 4/70 (5.7) (Pajares et al. 2018), 1/17 (5.9) (Macias 2016)	0.000017
	c.9018C>A	p.Tyr3006Ter	rs80359154	2/47 (4.3)	BC	7/155 (4.5) (Juan et al. 2013), 10/70 (14.3) (Pajares et al. 2018)	N/A
	c.9026_9030delATCAT	p.Tyr3009Serfs	rs80359741	2/47 (4.3)	BC (1), Ui (1)	33/155 (21.3) (Juan et al. 2013), 2/47 (4.3) (Rodríguez-Balada et al. 2020), 1/6 (16.7) (Hernan et al. 2015)	N/A
	c.1608dupT	p.Glu537Terfs	rs276174811	1/47 (2.1)	BC	–	N/A
	c.1128delT	p.Phe376Leufs	rs80359263	1/47 (2.1)	BC	–	N/A
	c.1813dupA	p.Ile605Asnfs	rs80359306	1/47 (2.1)	BC	–	0.000026
	c.2197_2198ins(157)	p.Val733Glyfs*22	NOVEL	1/47 (2.1)	OC	–	N/A
	c.2376C>A	p.Tyr792Ter	rs80358503	1/47 (2.1)	BC	–	N/A
	c.2701delC	p.Ala902Leufs	rs397507637	1/47 (2.1)	OC	–	N/A
	c.3847_3848delGT	p.Val1283Lysfs	rs80359405	1/47 (2.1)	BC	–	0.00012
	c.4060dupA	p.Thr1354Asnfs*7	NOVEL	1/47 (2.1)	BC	–	N/A
	c.4263dupT	p.Glu1422Terfs	rs155528366464	1/47 (2.1)	BC	–	N/A
	c.4380_4381delTT	p.Ser1461Leufs	rs397507715	1/47 (2.1)	BC	–	N/A
	c.4740_4741dupTG	p.Glu1581Valfs	rs864622401	1/47 (2.1)	OC	–	N/A
	c.5669_5673delTGGCA	p.Met1890Argfs	rs876660311	1/47 (2.1)	BC	–	N/A
	c.5722_5723delCT	p.Leu1908Argfs	rs80359530	1/47 (2.1)	BC	1/7 (14.2) (Ortiz et al. 2016)	N/A
	c.6034delT	p.Ser2012Profs	rs397507823	1/47 (2.1)	BC	–	0.000008
	c.6209_6212delAAAG	p.Glu2070Valfs	rs276174866	1/47 (2.1)	OC	–	N/A
	c.7110dupA	p.Ser2371Ilefs	rs80359638	1/47 (2.1)	BC	–	N/A
	c.7618-1G>A	N/A	rs397507389	1/47 (2.1)	BC	–	N/A
	c.7863T>A	p.Tyr2621Ter	rs276174896	1/47 (2.1)	BC	–	N/A
	c.8487+1G>A	N/A	rs81002798	1/47 (2.1)	BC	–	N/A
	c.9117G>A	p.Pro3039 =	rs28897756	1/47 (2.1)	Ui	–	N/A
	c.9382C>T	p.Arg3128Ter	rs80359212	1/47 (2.1)	BC	1/17 (5.9) (Macias 2016)	0.000016
	c.9413T>G	p.Leu3138Ter	rs886040838	1/47 (2.1)	BC	–	N/A
*ATM*	c.320dupG	p.Cys107Trpfs*8	NOVEL	1/7 (14.3)	BC	–	N/A
	c.790delT	p.Tyr264Ilefs	rs587781978	1/7 (14.3)	BC	–	8 × 10^–6^
	c.2921+1G>A	p.Tyr947fs	rs587781558	1/7 (14.3)	BC	2/95 (2.1) (Fonfria et al. 2021)	0.00002
	c.3046G>T	p.Gly1016*	NOVEL	1/7 (14.3)	BC	–	N/A
	c.6215delG	p.Gly2072Aspfs*10	NOVEL	1/7 (14.3)	BC	–	N/A
	c.7751_7754delCTAA	p.Thr2584Lysfs	NOVEL	1/7 (14.3)	BC	–	N/A
	c.8122G>A	p.Asp2708Asn	rs587782719	1/7 (14.3)	BC	–	N/A
*PALB2*	c.2964delA	p.Val989Terfs	rs587781840	3/8 (37.5)	BC	1/1 (100) (Ramírez-Calvo et al. 2019)	N/A
	c.1653T>A	p.Tyr551Ter	rs118203997	3/8 (37.5)	BC (2), Ui (1)	1/131 (0.8) (Blanco et al. 2013)	N/A
	c.2257C>T	p.Arg753Ter	rs180177110	1/8 (12.5)	BC	1/3 (33.3) (Rodríguez-Balada et al. 2020), 1/4 (25) (Gutiérrez-Enríquez et al. 2014)	0.000033
	c.1535dupA	p.Tyr512*fs*1	NOVEL	1/8 (12.5)	BC	–	N/A
*CHEK2*	c.349A>G	p.Arg117Gly	rs28909982	3/6 (50)	BC (2), Ui (1)	2/3 (66.7) (Paulo et al. 2018), 44/297 (14.8) (Southey et al. 2016), 2/19 (10.5) (Petridis et al. 2019)	0.000132
	c.507delT	p.Phe169Leufs	rs587780183	1/6 (16.7)	BC	–	0.000008
	c.1427C>T	p.Thr476Met	rs142763740	1/6 (16.7)	BC	–	0.000379
	g.34850-34958_43301-43362dup	N/A	NOVEL	1/6 (16.7)	BC	–	N/A
*MSH6*	c.738_741delAAAA	p.Lys246Asnfs	N/A	1/2 (50)	BC	–	N/A
	c.2314C>T	p.Arg772Trp	rs63750138	1/2 (50)	Ui	–	0.000033
*RAD51C*	c.104dupC	p.Glu36*fs*1	NOVEL	1/2 (50)	BC	–	N/A
	g.614-747_1069-2562del	N/A	NOVEL	1/2 (50)	Ui	–	N/A
*TP53*	c.722C>G	p.Ser241Cys	rs28934573	1/2 (50)	OC	–	0.000008
	c.848G>A	p.Arg283His	rs371409680	1/2 (50)	BC	–	0.000066

A list of all pathogenic variants identified in this study (ordered by their frequency) is shown together with frequencies, calculated based on the total number of mutations identified per gene. Also, frequencies of recurrent variants previously detected in other studies of Spanish populations that coincide with ours are shown

*BC* breast cancer, *OC* ovarian cancer, *Ui* unaffected individuals, *N/A* non applicable, *ExAC* The Exome Aggregation Consortium

**Fig. 2:**
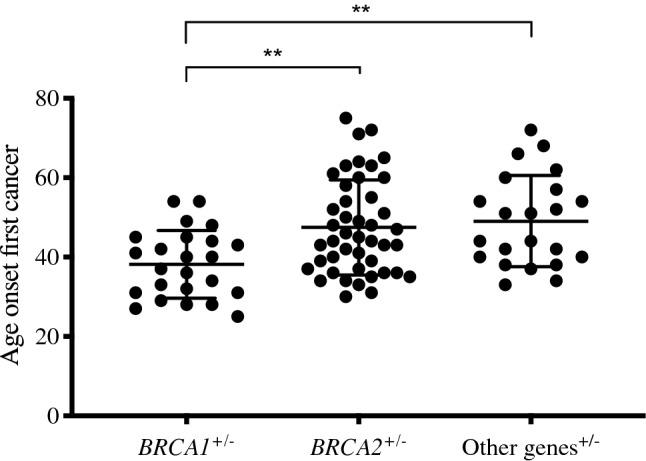
Age of onset of the first tumor grouped by gene variant. Scatter plots showing the age of cancer onset distribution accounting for the gene carrying heterozygous germline variant. *p* value for Kruskal–Wallis test is **p = 0.001

Interestingly, the age of onset of either BC or OC was significantly lower for *BRCA1* mutated patients compared to those carrying either *BRCA2* (*p* = 0.0013) or variants in other genes (*p* = 0.0012) (Fig. 2). In particular, the risk of presenting a pathogenic variant in the *BRCA1* gene (with respect to other genes) was reduced by 9% with each unit increase in the age at diagnosis (*p* = 0.002), being present in 92% of patients with estrogen receptor positive (ER +) (*p* < 0.001), and 89% of patients with progesterone receptor positive (PR +) (*p* < 0.001) and this significant effect was maintained when adjusting by age.

Regarding *BRCA1* and *BRCA2*, there were six and nine pathogenic mutations respectively that were identified more than once, suggesting their frequency might be high in the study population. Predictably, some of them had previously been found to be common in Spanish populations **(**Table 2).

Our cohort also includes 101 VUS which pathogenicity was studied using different *in-silico* predictors. Novel variants (11 VUS and 12 pathogenic) all of them, except for one, frameshifts not previously described were identified in our cohort, characterized and submitted to ClinVar (Table 2; SUB6135852). Two of the novel pathogenic variants were located at *BRCA2* and one at *BRCA1*. The remaining nine novel variants were located at *ATM* (4), *PALB2* (1), *CHEK2* (1), *MSH6* (1) and *RAD51C* (2). All patients carrying either of these novel variants developed cancer (except for one cancer-free individual) with only one case showing ovarian while the remaining 9 cases had breast cancer.

Regarding the clinical impact of the genetic variants identified in this study, we found that the risk of having a pathogenic or VUS variant in *MSH6* was reduced by 94% in patients ER + BC (*p* = 0.004) and 88% in patients PR + BC (*p* = 0.025) and this significant effect was maintained for age-adjustments. Furthermore, the risk of having a pathogenic or VUS variant in *PALB2* was increased by 6% with each unit increase in the age at diagnosis (*p* = 0.023). The risk of developing basal BC was increased 15.7 times in patients with *BRCA1* pathogenic variants (*p* < 0.001) and was reduced by 81% in those with *BRCA2* variants (*p* = 0.009). This effect was maintained when adjusting for age and also it was reproduced in multivariate logistic analyses including potentially confounding factors. The risk of developing HER2 positive BC was increased 37.5 times in individuals with *MSH6* pathogenic variants compared with variants in other genes (*p* = 0.031) and the risk of developing luminal A BC was reduced by 77% (*p* = 0.089) in patients with *BRCA1* pathogenic variants and this was maintained when adjusting by age. None of the pathogenic variants was significantly associated with luminal B BC. All these analyses were replicated including only women and no significant differences were observed (data not shown).

Considering clinical evolution, 6.1% (6/98) with a class IV/V variant died before the follow-up so they were excluded from this evaluation. Of them, three died due to cerebral metastases (two with BC before 40 and one with OC at 47) and other metastases for the remaining three.

More than a half of the patients (49/92) with a pathogenic or likely pathogenic variant underwent risk-reducing prophylactic surgery. We observed significant differences in the effect of the age at which cancer was diagnosed and the prophylactic clinical decisions undertaken (*p* = 0.008), with younger ages deciding to accept a mastectomy alone (median age 38.0) or in combination with oophorectomy (median age 35.5) compared to clinical follow-up alone (median age 44.5). The same effect was maintained if accounting for the age at which the genetic testing was performed with median ages of 43.1 and 39.9 for those undertaking mastectomy alone or in combination with oophorectomy respectively in comparison with those not deciding to undertake prophylaxis with median age of 55.0 (*p* < 0.001). However, no differences were observed among the age at which the prevention measures were taken (*p* = 0.342). Specifically, 47.0% underwent prophylactic oophorectomy, 36.7% underwent a prophylactic mastectomy, and 16.3% underwent both surgical procedures. Most of them carried a variant in *BRCA2* (27/49), followed by *BRCA1* (14/49). In addition, 8 patients opted for surgery carrying class IV/V variants in genes different from *BRCA1* or *BRCA2*, specifically 4 patients with *PALB2* variants (two with c.2964delA, and one with c.2257C>T or c.1535dupA) and 4 patients with a variant in either *ATM* (c.8122G>A), *CHEK2* (c.349A>G), *RAD51C* (c.104dupC) or *MSH6* (c.738_741delAAAA) respectively. Interestingly, only 14.3% of the patients carrying a pathogenic/likely pathogenic variant in *ATM* opted for prophylaxis (95% IC [1.97–58.1%] (*p* = 0.042).

## Discussion

Analysis of additional genes besides *BRCA1/2* determines the originality of this work, increasing the proportion of class IV/V variants identified in our cohort compared with previous studies in our region (Pajares et al. 2018). Detection rate of these variants was greater than the recommended 10% for hereditary cancer (Graña et al. 2011) highlighting efficiency of both cohort selection and genetic testing technology.

Frequency of pathogenic variants identified in this study (11.9%) is within the range of previous reports (Gabaldó Barrios et al. 2017; Pajares et al. 2018; del Manzanares Campillo et al. 2018; Blanco et al. 2013; Díez et al. 2003) although mutation frequencies for *BRCA1/2* in Spain were reported to vary from 6.8 (Beristain et al. 2007) to 33.3% (Miramar et al. 2008). Discrepancies among studies might be due to several reasons, being the inclusion criteria one of the main sources. For example, one of the studies with greater mutation frequencies for *BRCA1/2* (33.3%) included not only index patients but also their relatives, increasing the chances of detecting variants if we take into account that all these patients belonged to families with several breast/ovarian cancers (Miramar et al. 2008). Contrarily, a study showing very low mutation frequencies for *BRCA1/2* included only unrelated index cases with or without a family history of breast and/or ovarian cancer (Beristain et al. 2007) reducing the likelihood of detecting variants in these genes. Also, founder mutations might affect differential mutation frequencies across regions as shown previously (Díez et al. 2003) and population size also impacts the ability of detecting cancer-predisposing mutations.

**Fig. 3:**
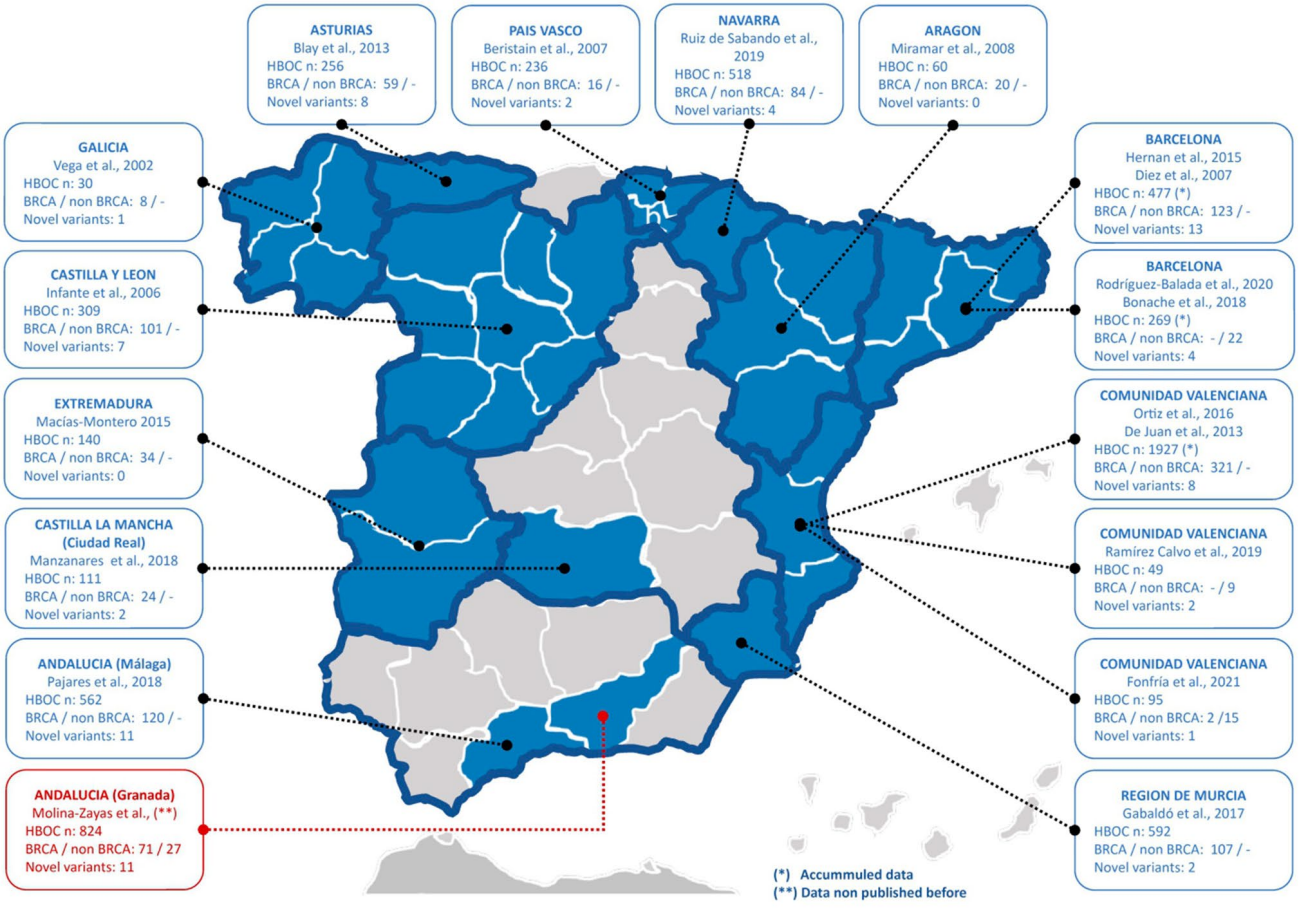
Graphical representation of the previous genetic testing studies in hereditary breast and ovarian cancer patients in Spain by regions. Number of individuals included in each cohort is shown with an "*n*". The number of pathogenic/likely pathogenic variants identified is also shown splitting by those studies including only *BRCA1/2* or other genes and the number of novel variants identified is also shown for comparison with our study

We identified higher mutation frequencies in *BRCA2* (48.0%) compared to *BRCA1* (24.5%) in line with recent studies (Pajares et al. 2018; del Manzanares Campillo et al. 2018); however, other authors found greater frequencies in *BRCA1* vs *BRCA2* (Gabaldó Barrios et al. 2017; Díez et al. 2003). This differential prevalence might be affected by study population size, inclusion criteria or even region-bias (Fig. 3).

Furthermore, contrary to most studies in our region, we analyzed not only *BRCA1/2* prevalence but also other HBOC risk-related genes. In our cohort, *PALB2* was the most commonly mutated gene (2.1%) after *BRCA1/2* validating previously reported frequencies (≈ 1%) (2013; Thompson et al. 2016), with two variants (c.1653T>A and c.2964delA) being found in three times each. Frequency of pathogenic variants in *ATM* (1.8%) also agreed with previously reported frequencies for Spanish populations (2%). However, frequencies of pathogenic variants in *CHEK2* (1.6%) and *TP53* (0.5%) in our cohort were slightly lower than previously reported (Walsh et al. 2006; Cipriano et al. 2018).

Data from NGS highlighted a reduction in the number of low-risk reported individuals (based on the presence of class I/II variants) from 85.4 to 65% (for targeted and NGS respectively) suggesting that the inclusion of additional genes will improve the detection of high-risk individuals. Importantly, this reduction came with an increase in VUS that need to be re-classified on a periodic basis. Importantly, 27 individuals in this cohort would have been misclassified as low-risk if a gene panel had not been carried out. In fact, it was recently demonstrated that a significant proportion of BC patients with germline variants do not meet classical NCCN testing criteria, suggesting that NGS might be used in the near future for screening less targeted populations as the costs fall.

Prophylactic mastectomy (bilateral and contralateral) is one of the most widely used options to reduce cancer risk in women who are carriers of a pathogenic/likely pathogenic variant in risk genes, being able to reduce more than 85% of the incidence of breast cancer. Oophorectomy is known as the surgical procedure of removing adnexal organs (ovary and fallopian tubes) unilaterally or bilaterally in women who are carriers of pathogenic *BRCA1* and *BRCA2* variants. Most of the published articles refer to risk-reducing surgeries based on pathogenic variants in these two genes, however, in our cohort, 8 of the women who underwent risk-reducing surgery carried a variant in genes such as *PALB2, ATM, CHEK2, ATM* and *MSH6*. Even though there are no many guidelines on prophylactic surgery beyond *BRCA1* and *BRCA2*, the number of oncologists and gynecologists recognizing the risk of developing HBOC associated with other high or intermediate penetrance genes is rising. Recently, the ACMG published a guideline for patients with germline variants in *PALB2*, in which risk-reducing mastectomy is an option to be considered to reduce BC risk as well as to include surveillance for pancreatic cancer; however, oophorectomy is not recommended for patients below 50 years. In our cohort, 3 out of 8 patients with class IV/V variants in this gene undertook prophylactic mastectomy as recommended by this recent guideline. Furthermore, we found that younger women at diagnosis (or genetic testing) were more likely to undergo prophylactic measures although the likelihood of selecting these preventive measures was reduced by 87% in patients with a pathogenic/likely pathogenic variant in *ATM*.

In addition, it is important to take into account other factors such as women's age at the time of clinical diagnosis of cancer and age at the time of the genetic diagnosis. This is of great importance since we demonstrated that women at younger ages preferably chose to undergo surgery. On the other hand, the family cancer history should also be considered as well as the psychological aspects such as fear of cancer recurrence or its association with the development of other cancers depending on the mutated gene (pancreas, endometrium, Cowden syndrome, stomach, etc.). To our knowledge, this is the first time that NGS including non-*BRCA* genes was performed in an Andalusian cohort to assess germline variants predisposing to HBOC. Our data agrees with previous studies for targeted *BRCA1/2* variants in high-risk HBOC individuals and reduces the number of class I/II reported variants validating the use of NGS to increase the likelihood of variant identification.

## Supplementary Information

Below is the link to the electronic supplementary material.

Supplementary file1 (DOCX 13 KB)

Supplementary file2 (XLSX 82 KB)

## Data Availability

Novel pathogenic/likely pathogenic/VUS variants identified in this study were reported to ClinVar under submission SUB6135852. All pathogenic/likely pathogenic variants identified are shown in Table 2 with rs numbers to allow their identification in public databases.
