# Supplementary material for: Identification of hereditary breast and ovarian cancer germline variants in Granada (Spain): NGS perspective
Source: Mol Genet Genomics. 2022 Apr 22;297(3):859–71. doi: 10.1007/s00438-022-01891-5 (PMC9130174; doi:10.1007/s00438-022-01891-5)
Supplement: Supplementary file 1 — Supplementary file1 (DOCX 13 KB) [file 438_2022_1891_MOESM1_ESM.docx]

**Supplementary table 1**: Inclusion criteria for the study cohort defined according to the Spanish Society of Clinical Oncology (SEOM). Abbreviations are: BC=Breast Cancer; OC=Ovarian Cancer

| n | Clinico-pathological characteristics |
| --- | --- |
| 1 familiar cancer (independent on family history) | - Synchronic or metachronic breast (BC) and ovarian (OC) cancer in the same individual.  - BC diagnosed before 35 years old.  - Bilateral BC, when the first was diagnosed before 40 years old.  - Triple negative BC diagnosed before 50 years old.  - High-grade serous papillary OC. |
| 2 familiar cancers (first degree relatives and in the same family branch) | - Bilateral BC diagnosed before 50 years old.  - 1 BC in a male and BC/OC in a female of the family.  - BC and OC.  - 2 BC diagnosed before 50 years old. |
| ≥3 BC and/or OC in the family | ≥3 BC and/or OC in the family (independently on age). |
